# Temporal trend of violence due to neglect/abandonment in older women in Brazil (2009-2022)

**DOI:** 10.1590/0034-7167-2025-0379

**Published:** 2026-09-28

**Authors:** Beatriz Caroline Leão Lima, Marijany Duarte da Silva Reis, Ana Larissa Gomes Machado, Ana Maria Ribeiro dos Santos, Francisca Tereza de Galiza, Francisco Gilberto Fernandes Pereira, Lívia Carvalho Pereira, José Wicto Pereira Borges

**Affiliations:** I Universidade Federal do Piauí. Teresina, Piauí, Brazil

**Keywords:** Elder Abuse, Aged, Comprehensive Health Care, Health of the Elderly, Temporal Distribution., Abuso de Ancianos, Anciano, Atención Integral de Salud, Salud del Anciano, Distribución Temporal.

## Abstract

**Objectives:**

to analyze the temporal trend of reported cases of violence due to neglect/abandonment against older women in Brazil between 2009 and 2022.

**Methods:**

an ecological time-series study and spatial analysis using data from the Notifiable Diseases Information System. Prais-Winsten regression was used to estimate the Annual Percentage Change (APC) in violence rates among women aged 60 or older according to Brazilian regions.

**Results:**

34,860 cases were recorded, with the highest occurrence in the Southeast and a more pronounced proportional growth in the Northeast (APC: 31.48%). Most episodes occurred in the home (80.2%), with offenders being a family member in 67.5% of cases, and 42% of victims reported repeated violence.

**Conclusions:**

a growing and uneven trend was observed in reports of violence due to neglect and abandonment against older women in Brazil, highlighting the persistence of this violence.

## RESUMEN

**Objetivos:**

analizar la tendencia temporal de los casos notificados de violencia por negligencia/abandono contra mujeres mayores en Brasil entre 2009 y 2022.

**Métodos:**

estudio ecológico de series temporales y análisis espacial con datos del Sistema de Información de Enfermedades de Declaración Obligatoria. Se utilizó la regresión de Prais-Winsten para estimar el Cambio Porcentual Anual (CPA) en las tasas de violencia entre mujeres de 60 años o más, según las regiones brasileñas.

**Resultados:**

se registraron 34.860 casos, con la mayor incidencia en el sureste y un crecimiento proporcional más pronunciado en el noreste (CPA: 31,48%). La mayoría de los episodios ocurrieron en el hogar (80,2%), siendo el agresor un familiar en el 67,5% de los casos, y el 42% de las víctimas reportaron violencia reiterada.

**Conclusiones:**

se observó una tendencia creciente y desigual en las denuncias de violencia por negligencia y abandono contra mujeres mayores en Brasil, destacando la persistencia de esta violencia.

## INTRODUCTION

Population aging is a global demographic phenomenon that is distributed in different proportions across various countries according to economic, environmental, social, and cultural conditions. It is noteworthy that in Latin America and the Caribbean, this process is occurring at a more accelerated rate^(1)^.

Projections indicate that the number of people aged 65 and over worldwide will more than double, rising from 761 million in 2021 to 1.6 billion in 2050. The number of people aged 80 and over is growing even faster^(2)^. In Brazil, the number of older adults identified in the last demographic census was 32,113,490, of which approximately 17.5 million are women, and about 4 million of them live in the Northeast region of the country^(3)^.

Research on older women’s lives indicates that this is a phase in which several factors combine to make them more vulnerable, such as: hormonal changes caused by menopause and their consequences on physical health; risk of depression and body image disorders^(4)^; economic losses; reduced autonomy in making decisions about one’s own body; and vulnerability to situations of violence and abandonment^(5)^.

Neglect or abandonment is understood to be violence that occurs when there is an omission or refusal to provide necessary care, which can result in significant harm to older adults’ physical and psychological well-being^(6)^. This type of violence is the second most frequent in northeastern Brazil (17.3%), second only to physical violence (28.8%)^(7)^.

Violence stemming from neglect or abandonment of older adults is multifaceted and multidimensional, with diverse social and individual consequences, constituting a public health problem. It is one of the most common forms of violence in this population, despite being the least reported.

When considering gender, this type of violence can present differences in its trends and spatial distribution among older women, with patterns that need to be understood by health systems so that more effective public policies can be developed to combat and prevent it.

International and national studies demonstrate that elder abuse is a highly prevalent phenomenon. They indicate that approximately one in six older people suffer some type of abuse, with neglect being one of the most common and silent forms, especially among women in contexts of greater social vulnerability. Furthermore, they highlight that elder abuse is of alarming magnitude, with a predominance of cases of neglect and abandonment perpetrated by family caregivers^(8,9)^. These findings reflect the burden of informal care and the fragility of social and institutional support networks aimed at older adults.

Despite these advances, the national literature on elder abuse is still limited. Most studies present local snapshots or short periods, without considering the temporal evolution of reports on a national scale.

Considering that data synthesis regarding elder abuse is scarce, especially in middleand low-income countries^(10)^, the need for investigations that address this topic in a more in-depth way, with systematic and longitudinal analysis, considering regional specificities and the recent sociopolitical context for violence due to neglect and abandonment against older women, becomes evident.

## OBJECTIVES

To analyze the temporal trend of reported cases of violence due to neglect/abandonment against older women in Brazil between 2009 and 2022.

## METHODS

### Ethical aspects

There was no need to submit the data to the Research Ethics Committee, as publicly accessible secondary data were used, guaranteeing the anonymity of all participants whose data were analyzed.

### Study design, period, and location

This is a mixed ecological study, using time series and spatial analysis, based on reported cases of violence through abandonment and/or neglect against older women in Brazil. The study was conducted in accordance with the STrengthening the Reporting of OBservational studies in Epidemiology guideline, proposed by the EQUATOR Network for reporting observational studies.

The period analyzed encompassed the years 2009 to 2022, covering the entire national territory, with stratification according to the five major Brazilian geographic regions (North, Northeast, Southeast, South, and Midwest).

Thus, Brazil has an approximate territorial extension of 8.5 million km^2^ and an estimated population of around 203 million inhabitants. Women make up approximately 51% of the Brazilian population, with those aged 60 or older accounting for approximately 8.1% of the country’s female population^(3)^.

### Population or sample; inclusion and exclusion criteria

The study population consisted of all records of elder abuse, aged 60 years or older, reported in the Notifiable Diseases Information System (In Portuguese, *Sistema de Informação de Agravos de Notificação* - SINAN) between 2009 and 2022.

Reports concerning older women whose type of violence was classified as neglect and/or abandonment were included. Records lacking information about the victim’s sex, as well as those where the type of violence was not specified, were excluded.

### Study protocol

The study was conducted using secondary data obtained from information provided by the Brazilian Unified Health System Department of Informatics (In Portuguese, *Departamento de Informática do Sistema Único de Saúde* - DATASUS)^(6)^. The number of cases of violence due to neglect and/or abandonment against older women was extracted from SINAN, while population estimates were obtained from the Brazilian Institute of Geography and Statistics (In Portuguese, *Instituto Brasileiro de Geografia e Estatística* - IBGE). Both databases are available on the Ministry of Health’s DATASUS website.

Data were extracted, organized, and exported to Microsoft Excel^®^, where descriptive analyses were performed. For temporal trend analysis, Stata^®^ version 14.0 was used.

The following variables were considered: female sex; age group equal to or greater than 60 years; geographic region; federative unit; municipality; year of reporting; kinship with the offender; violence location; and occurrence of repeated violence.

To calculate the gross reporting rates, the total number of cases of violence due to neglect and/or abandonment against older women was divided by the corresponding resident population, and the quotient was multiplied by 100,000 inhabitants.

### Statistical and time trend analysis

The analysis of the temporal trend of gross rates of violence due to abandonment and/or neglect against older women was performed using Prais-Winsten generalized linear regression, considering a significance level of 5% (p<0.05)^(11)^. This method was adopted because it allows for the correction of serial autocorrelation of residuals in time series.

The dependent variable (Y) was defined as the gross rate of reported cases of violence due to abandonment and/or neglect against older women, while the independent variable (X) corresponded to the year of reporting. From the slope coefficient (*b1*) estimated by the model, the Annual Percentage Change (APC) was calculated, used to classify the temporal trend as upward (positive APC), downward (negative APC), or stationary, when there was no statistically significant difference from zero (p>0.05). The dependent variable (Y) was the gross rate of violence due to abandonment and/or neglect of older women, and the independent variable (X) was the year of reporting^(11)^.

Considering the potential impact of the COVID-19 pandemic on violence records, analyses were conducted over two distinct periods - 2009-2019 and 2009-2022 -, allowing for an assessment of the influence of including this event on temporal trends. To minimize aggregation bias, also known as ecological fallacy, a stratified analysis plan was adopted based on the country’s major geographic regions and place of residence.

Following the method proposed by Bottomley *et al*.^(11)^, initially, the base-10 logarithmic transformation of the rates (Y) was performed in order to reduce the heterogeneity of the variance of the residuals. Then, the Prais-Winsten model was applied to estimate the coefficient *b1*, from which the APC was calculated according to the formula: APC= [-1 + 10^b^_1_] *100%.

The 95% Confidence Intervals (95%CI) for APC were calculated using the limits of the *b*1 coefficient estimated by the model, according to the expression: 95%CI= [-1+10^b^_1_^minimum^] *100; [-1+10^b^_1_^maximum^] *100. The minimum and maximum *b_1_* values are the limits of the Confidence Interval (CI) generated by the statistical analysis program. They were applied to the formula, with the minimum *b_1_* value corresponding to the minimum point of the CI, and the maximum *b_1_* value corresponding to the maximum point of the CI. In this study, the significance level considered was 5% for all statistical tests.

## RESULTS

The magnitude of cases of neglect/abandonment violence recorded between 2009 and 2022 was 34,860. It is noteworthy that, in the years corresponding to the COVID-19 pandemic, more than 33% of cases in the entire time series were recorded. In terms of region, the Southeast region recorded the highest percentage of cases in the analyzed periods, followed by the Northeast and South regions.

Table 1 presents the characteristics of older women who were victims of this type of violence according to their relationship with the offender, incident location, and whether the violence was repeated. Among the reported cases, in more than 67%, offenders had a family relationship with the victim. The home was the most frequent location (>80%), indicating abandonment/neglect in their own place of residence, with an increase of more than 34% in the event occurrence during the COVID-19 pandemic. Repeated violence occurred in more than 40% of the older women studied during the period.

**Table 1:** Characterization of reported cases of neglect/abandonment of older women in Brazil (2009-2022), Teresina, Piauí, Brazil, 2025

Variables	2009-2019		2009-2022	
	n	(%)	n	(%)
**Occurrence of violence due to neglect/abandonment**				
Brazil	22,493	100	34,860	100
North	534	2.37	762	2.19
Northeast	5,227	23.24	9,527	27.33
Southeast	8,427	37.46	12,985	37.25
South	5,188	23.06	7,451	21.37
Midwest	3,112	13.84	4,135	11.86
**Kinship**				
Family^*^	15,128	67.26	23,512	67.45
Affectionate^**^	1,145	5.09	1,682	4.83
Acquaintance^***^	2,218	9.86	3,071	8.81
Unknown	215	0.96	298	0.85
Person with an institutional relationship	320	1.42	476	1.37
Police officer/law enforcement agent	15	0.07	18	0.05
Other ties	3,456	15.36	5,017	14.39
**Occurrence location**				
Residence	18,239	81.09	27,943	80.16
Collective housing	315	1.40	505	1.45
School	5	0.02	7	0.02
Sports facility	9	0.04	12	0.03
Bar or similar establishment	4	0.02	10	0.03
Public road	516	2.29	827	2.37
Commerce/services	293	1.30	515	1.48
Industry/Construction	5	0.02	7	0.02
Other	1,186	5.27	1,834	5.26
**Repeated violence**				
Yes	10,160	45.17	14,546	41.73
No	4,098	18.22	7,203	20.66

* Father, mother, stepfather, stepmother, son/daughter, brother/sister;

** Spouse, ex-spouse, boyfriend/girlfriend, ex-boyfriend/girlfriend;

*** Friends/acquaintances, caregiver, boss/employer.

*Source: Brazilian Unified Health System Notifiable Diseases Information System/Information Technology Department.*

Table 2 presents a temporal analysis of cases of violence due to neglect/abandonment of older women in Brazil and by region. Brazil showed a statistically significant increasing trend for both periods analyzed (APC: 20,23; 95%CI 10,71; 30,57; p-value: <0001; APC: 16,76; 95%CI: 8,62; 25,51; p-value: <0,001). Among the regions, considering the period from 2009 to 2019, the APC was higher in the North regions (APC: 43,13; 95%CI: 17,73; 74,00; p-value: 0,002) and Northeast (APC: 38,50; 95%CI: 20,05; 59,79; p-value: <0001). Between 2009 and 2022, the Northeast region showed the highest APC (APC: 31,48; 95%CI: 17,86; 46,67; p-value: < 0,001), followed by the North region (APC: 29,58; 95%CI: 8,55; 54,68; p-value: 0,008). The Midwest region showed a stationary trend for both periods analyzed (APC: 8,22; 95%CI: -1,82; 19,29; p-value: 0,100; APC: 4,47; 95%CI: -3,07; 12,59; p-value: 0,228). No reduction in violence rates was observed in any country region.

**Table 2:** Annual Percentage Change in gross rates (per 100,000 inhabitants) of violence related to neglect/abandonment among older adults in Brazil (2009-2022), Teresina, Piauí, Brazil, 2025

Region	Period	APC	95%CI	*p* value	DW	Trend
North	2009-2019	43.13	17.73; 74.00	0.002	1.62	Upward
	2009-2022	29.58	8.55; 54.68	0.008	1.83	Upward
Northeast	2009-2019	38.50	20.05; 59.79	0.001	1.36	Upward
	2009-2022	31.48	17.86; 46.67	< 0.001	1.33	Upward
Southeast	2009-2019	15.86	9.93; 22.11	< 0.001	1.61	Upward
	2009-2022	12.72	7.21; 18.51	< 0.001	1.64	Upward
South	2009-2019	30.95	9.09; 57.18	0.009	1.13	Upward
	2009-2022	22.48	4.76; 43.19	0.015	1.14	Upward
Midwest	2009-2019	8.22	-1.82; 19.29	0.100	1.04	Stationary
	2009-2022	4.47	-3.07; 12.59	0.228	1.23	Stationary
Brazil	2009-2019	20.23	10.71; 30.57	0.001	1.09	Upward
	2009-2022	16.76	8.62; 25.51	0.001	1.17	Upward

*APC - Annual Percentage Change; 95%CI - 95% Confidence Interval; DW - Durbin-Watson.*

*Source: Brazilian Unified Health System Notifiable Diseases Information System/Information Technology Department.*

Figure 1 shows the evolution of the gross rate of reported cases of violence due to abandonment/neglect of older women by region from 2009 to 2022. It can be observed that, in the Midwest region, the reporting of this violence increased from 2009 to 2013, with a period of decline and fluctuations in the remaining years. The Northeast region showed growth throughout the period, with an increase in reports during the COVID-19 pandemic. The South and Southeast regions showed similar behaviors during the period. The North region showed the lowest growth rate, with a peak in reports between 2015 and 2017.

**Figure 1:**
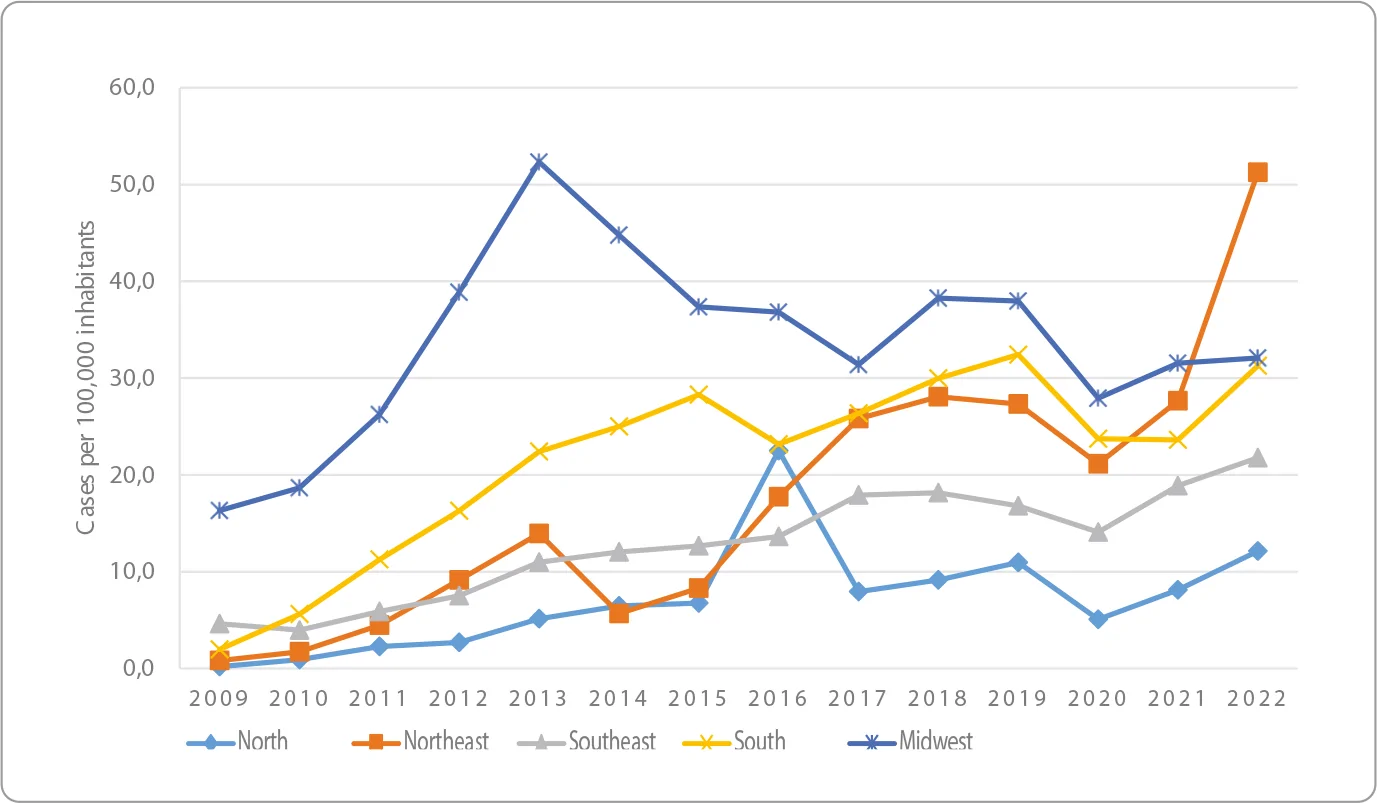
Gross rate of reported cases of violence related to neglect/abandonment among older adults in Brazil by region (2009-2022), Teresina, Piauí, Brazil, 2025

*Source: Brazilian Unified Health System Notifiable Diseases Information System/Information Technology Department.*

## DISCUSSION

The results show a temporal trend of increasing cases of violence due to neglect/abandonment of older women in Brazil, especially during the years encompassing the peak of the COVID-19 pandemic, both in the overall data and by country region. Despite the magnitude of the issue and the serious consequences for physical and mental health, academic production on the subject is still incipient worldwide^(12)^. In Brazil, the Federal Government made its first statement about the increase in elder abuse only four months after the first confirmed case of the disease in the country^(13)^. This increase can be attributed to multiple factors, including social isolation, overburdened care services, and greater socioeconomic vulnerability among older people.

In this context, it is worth highlighting that violence against women tends to increase during emergencies of any kind, including epidemics, considering the breakdown of social networks and more restricted access to health and social protection services^(14)^. Although scarce at the start of the COVID-19 pandemic, reports in France, China, and the United States have suggested an increase in cases of domestic violence since the beginning of the outbreak^(15)^.

A study conducted in Africa highlights the increased risk of elder abuse by their caregivers during the COVID-19 pandemic^(16)^. This phenomenon is revealed to be worrying in the data from the study in question, which points to the occurrence of abandonment/neglect in older woman’s own place of residence, with the violence being perpetrated mainly by someone with a family relationship.

This creates a scenario of domestic elder abuse, already evidenced in other national studies, but intensified by social isolation resulting from measures to control SARS-CoV-2 contamination. In 2020, The Guardian newspaper reported a global increase in cases of domestic violence. Brazil experienced a 40% to 50% increase, and Spain a 20% increase, in the number of calls to the “Dial 100” helpline during the first days of lockdown^(17)^.

Furthermore, a pattern was observed that is repeated globally in which violence against women is prevalent and perpetrated by intimate partners and, in the case of older adults, by family caregivers. The situation becomes even more harmful when considering that the violence is suffered in silence, and ends up being covered up by the proximity and dependence between the offender and the victim or by the fear of retaliation and/or abandonment^(13)^.

Furthermore, a high number of reported cases of repeated violence were observed for both periods analyzed in this study, revealing a serious increase in the vulnerability of older women who are sometimes victims of isolation and abandonment by family members.

This care, sometimes associated with the idea of familism - a multidimensional construct that emphasizes the family as primarily responsible for the support and care of its members - can become a vulnerability factor when there is no effective support from social policies and assistance services. Such a scenario can favor the occurrence of domestic violence^(18)^.

Therefore, emotional and financial dependence, fear, difficulties in accessing social services, and the normalization of violence throughout life contribute to the invisibility of this problem^(10)^.

Violence and abuse suffered by older adults can also trigger anxiety and depressive symptoms, contributing to social isolation and feelings of loneliness^(19)^. When this reality is analyzed from a gender perspective, it is observed that older women have a higher prevalence of these health problems compared to men, highlighting an even more pronounced vulnerability that constitutes a public health issue^(4)^.

In spatial analysis, the evolution of the gross rate of reported cases of violence due to abandonment/neglect of older women shows that northeastern Brazil experienced growth throughout the analyzed period, as indicated in results from previous time series studies. The Midwest region showed fluctuations during the period, and the North region presented the lowest growth rates. The South and Southeast regions showed similar behaviors during the analysis period.

The Ministry of Human Rights and Citizenship reports that, from January to May 2023 alone, the Brazilian National Human Rights Ombudsman, through the “Dial 100” hotline, received 47,000 complaints of elder abuse throughout Brazil, registering 282,000 violations. In cases of neglect and abandonment, 37,400 violations of neglect and 19,900 of abandonment were reported, an increase of 855% compared to the previous year^(20)^.

Furthermore, the study developed by Camacho *et al*.^(21)^, which analyzed reports of elder abuse between 2020 and 2023, showed that the highest rate by region in Brazil was in the Southeast region, presenting 54.63% in 2020, 54.02% in 2021, 54.38% in 2022 and 53.54% in 2023. The Northeast region is in second place, with 20.09% of reports in 2020, 21.33% in 2021, 19.93% in 2022, and 19.90% in 2023. Next in line are the Midwest, North, and finally, the South regions. In all the years studied, older women consistently account for the largest percentage, accounting for 69.22% of all victims of violence, with the highest percentage in 2022.

Elder abuse in Brazilian regions is not uniform. Socioeconomic, cultural and access to protection services factors, which vary between regions, must be considered, impacting the prevalence and typology of aggressions. In this context, Andrade *et al*.^(22)^ investigated this profile from the perspective of urgency and emergency through the VIVA Survey of 2017. The study identified that males experienced the highest rate of physical assault by a stranger. Among older women, however, the data highlighted violence perpetrated by a partner, with threats being one of the most frequent forms of aggression.

Therefore, it is necessary for public policies to be adjusted to regional specificities, considering factors such as infrastructure, access to healthcare services, and socioeconomic vulnerabilities, in order to combat and prevent elder abuse. Furthermore, it is necessary to combat ageism and sexism when directly associated with older women, given that gender is a considerable risk factor^(23,24)^.

Given this scenario, it is recommended to expand strategies to combat elder abuse, including awareness campaigns, improved training for healthcare and social assistance professionals, and strengthening reporting and support channels. Future studies could further analyze the social determinants and psychosocial factors that contribute to the perpetuation of this type of violence, as well as explore effective interventions for its prevention and mitigation.

### Study limitations

The limitation relates to the possibility of underreporting of cases of neglect/abandonment violence, especially in northern and northeastern Brazil. This factor may compromise data accuracy and limit the generalizability of results. Since these are secondary data, there is a dependence on the information completeness and consistency in the reporting system. Furthermore, the categorization of neglect and abandonment violence may not encompass the full complexity of the social and cultural interactions that permeate this form of violence.

### Contributions to nursing, health, or public policy

This study broadens the understanding of the regional distribution and patterns of neglect against older women, offering technical and scientific support for nursing professionals to plan care strategies that are more sensitive to situations of vulnerability. In terms of public health, it highlights the need to strengthen ongoing training focused on early detection and addressing violence.

In the field of public policy, the findings reinforce the urgency of developing intersectoral actions that prioritize older adults’ safety, care, and rights in the home context, such as continuous training for caregivers and family members for home care, strengthening referral centers, and expanding community and intergenerational support networks aimed at creating protective bonds and reducing the isolation of older people. Such strategies favor the implementation of policies for active, participatory, and safe aging.

Therefore, this research contributes to the understanding of elder abuse in Brazil. The increasing number of reported cases highlights the urgency of concrete actions that guarantee the right to dignity, safety, and quality of life for this population.

## CONCLUSIONS

This study achieved its objective by demonstrating a growing temporal trend in reports of violence due to neglect and abandonment against older women in Brazil between 2009 and 2022, with particular emphasis on the period of the COVID-19 pandemic. A higher absolute number of reports was found in the Southeast region and a higher percentage of cases in the Northeast, highlighting significant regional inequalities.

The predominance of cases in home environments and family members’ involvement as the main offenders reveal the silent and persistent nature of this form of violence, often rendered invisible by healthcare services. These findings reinforce the need to strengthen surveillance, early detection, and continuous follow-up actions within Primary Health Care, with nursing playing a leading role in the care and qualified listening of families.

It is recommended to expand training strategies for caregivers and healthcare professionals, improve reporting procedures, and strengthen coordination between health, social assistance, and justice services. These actions are fundamental for effectively addressing neglect and abandonment, as well as for consolidating public policies aimed at promoting active, dignified, and protected aging, in line with Decade of Healthy Aging in the Americas (2021-2030) guidelines.

## Data Availability

The research data are available only upon request.
