# Supplementary material for: Temporal trend of violence due to neglect/abandonment in older women in Brazil (2009-2022)
Source: Rev Bras Enferm. 2026 Sep 28;79:e20250379. doi: 10.1590/0034-7167-2025-0379 (PMC13623390; doi:10.1590/0034-7167-2025-0379)
Supplement: Translation

# Translation: Tendência temporal da violência por negligência/abandono em mulheres idosas do Brasil (2009-2022)

**DOI:** 10.1590/0034-7167-2025-0379pt

**Authors:** Beatriz Caroline Leão Lima, Marijany Duarte da Silva Reis, Ana Larissa Gomes Machado, Ana Maria Ribeiro dos Santos, Francisca Tereza de Galiza, Francisco Gilberto Fernandes Pereira, Lívia Carvalho Pereira, José Wicto Pereira Borges

**Affiliations:** I Universidade Federal do Piauí. Teresina, Piauí, Brasil

**Keywords:** Abuso de Idosos, Idoso, Assistência Integral à, Saúde, Saúde do Idoso, Distribuição Temporal.

## Abstract

**Objetivos:**

analisar a tendência temporal das notificações de violência por negligência/abandono em mulheres idosas no Brasil entre 2009 e 2022.

**Métodos:**

estudo ecológico de série temporal e análise espacial que utilizou dados do Sistema de Informação de Agravos de Notificação. Utilizou-se regressão de Prais-Winsten para estimar a Variação Percentual Anual (VPA) das taxas de violência em mulheres com 60 anos ou mais segundo regiões brasileiras.

**Resultados:**

foram registrados 34.860 casos, com maior ocorrência no Sudeste e crescimento proporcional mais acentuado no Nordeste (VPA: 31,48%). A maioria dos episódios ocorreu no domicílio (80,2%), com agressor familiar em 67,5% dos casos, e 42% das vítimas relataram violência de repetição.

**Conclusões:**

observou-se tendência crescente e desigual das notificações de violência por negligência e abandono contra mulheres idosas no Brasil, evidenciando a persistência dessa violência.

## INTRODUÇÃO

O envelhecimento populacional é um fenômeno demográfico global que se distribui em diferentes proporções nos diversos países de acordo com as condições econômicas, ambientais, sociais e culturais, destacando-se que, na América Latina e Caribe, esse processo está ocorrendo de forma mais acelerada^(1)^.

A projeção é que o número de pessoas com 65 anos ou mais no mundo mais que dobre, passando de 761 milhões em 2021 para 1,6 bilhão em 2050. O número de pessoas com 80 anos ou mais está crescendo ainda mais rápido^(2)^. No Brasil, o número de idosos identificado no último censo demográfico foi de 32.113.490 pessoas, das quais aproximadamente 17,5 milhões são mulheres e cerca de 4 milhões delas vivem na região Nordeste do país^(3)^.

Pesquisas realizadas sobre o contexto de vida da mulher idosa apontam que esta é uma fase em que se somam alguns fatores que a tornam mais vulnerável como: as mudanças hormonais provocadas pelo climatério e suas consequências na saúde física; risco de depressão e distúrbios da autoimagem^(4)^; perdas econômicas; redução na autonomia de decisões sobre o próprio corpo; e vulnerabilidade a situações de violência e abandono^(5)^.

Entende-se que a violência por negligência ou abandono é aquela que ocorre quando existe omissão ou recusa da prestação dos cuidados necessários, podendo resultar em danos significativos para o bem-estar físico e psicológico da pessoa idosa^(6)^. Esse tipo de violência é o segundo mais frequente na região Nordeste brasileira (17,3%), perdendo apenas para a violência física (28,8%)^(7)^.

A violência por negligência ou abandono da pessoa idosa possui caráter multifacetado e multidimensional, com diversos desdobramentos sociais e individuais, configurando-se como um problema de saúde pública. É uma das formas mais comuns de violência nessa população, apesar de ser a menos denunciada.

No recorte de gênero, esse tipo de violência pode apresentar diferenças em sua tendência e distribuição espacial entre mulheres idosas, com padrões que necessitam ser compreendidos pelos sistemas de saúde, para que políticas públicas mais efetivas sejam construídas para o combate e a prevenção.

Estudos internacionais e nacionais demonstram que a violência contra pessoas idosas constitui um fenômeno de elevada prevalência. Apontam que cerca de uma em cada seis pessoas idosas sofre algum tipo de abuso, sendo a negligência uma das formas mais comuns e silenciosas, especialmente entre mulheres em contextos de maior vulnerabilidade social, além de destacar que a violência contra pessoas idosas dependentes assume magnitude preocupante, com predominância de casos de negligência e abandono perpetrados por familiares cuidadores^(8,9)^. Esses achados refletem a sobrecarga do cuidado informal e a fragilidade das redes de apoio social e institucional voltadas à população idosa.

Apesar desses avanços, a literatura nacional sobre violência contra a mulher idosa ainda é limitada. A maioria dos estudos apresenta recortes locais ou períodos curtos, sem contemplar a evolução temporal das notificações em escala nacional.

Considerando que a sintetização de dados no tocante à violência contra a mulher idosa é escassa, principalmente em países de renda média ou baixa^(10)^, torna-se evidente a necessidade de investigações que abordem essa temática de forma mais aprofundada, com análise sistemática e longitudinal, considerando as especificidades regionais e o contexto sociopolítico recente para a violência por negligência e abandono contra mulheres idosas.

## OBJETIVOS

Analisar a tendência temporal das notificações de violência por negligência/abandono contra mulheres idosas no Brasil no período de 2009 a 2022.

## MÉTODOS

### Aspectos éticos

Não houve necessidade de submissão ao Comitê de Ética em Pesquisa, pois foram utilizados dados secundários de acesso público, sendo garantido o anonimato de todos os participantes cujos dados foram analisados.

### Desenho, período e local do estudo

Trata-se de estudo ecológico misto, de série temporal e análise espacial, realizado a partir de casos notificados de violência por abandono e/ou negligência contra mulheres idosas no Brasil. O estudo foi conduzido de acordo com a diretriz *STrengthening the Reporting of OBservational studies in Epidemiology*, proposta pela Rede EQUATOR para o relato de estudos observacionais.

O período analisado compreendeu os anos de 2009 a 2022, abrangendo todo o território nacional, com estratificação segundo as cinco grandes regiões geográficas brasileiras (Norte, Nordeste, Sudeste, Sul e Centro-Oeste).

Desse modo, o Brasil possui extensão territorial aproximada de 8,5 milhões de km^2^ e população estimada em cerca de 203 milhões de habitantes. As mulheres correspondem a aproximadamente 51% da população brasileira, sendo que aquelas com idade igual ou superior a 60 anos representam cerca de 8,1% da população feminina do país^(3)^.

### População ou amostra; critérios de inclusão e exclusão

A população do estudo foi composta por todos os registros de violência contra pessoas idosas, com idade igual ou superior a 60 anos, notificadas no Sistema de Informação de Agravos de Notificação (SINAN) no período de 2009 a 2022.

Foram incluídas as notificações referentes a mulheres idosas cujo tipo de violência foi classificado como negligência e/ou abandono. Foram excluídos os registros que apresentavam ausência de informação sobre o sexo da vítima, bem como aqueles em que o tipo de violência não estava especificado.

### Protocolo do estudo

O estudo foi conduzido com base em dados secundários, obtidos a partir das informações disponibilizadas pelo Departamento de Informática do Sistema Único de Saúde (DATASUS)^(6)^. O número de casos de violência por negligência e/ou abandono contra mulheres idosas foi extraído do SINAN, enquanto as estimativas populacionais foram obtidas junto ao Instituto Brasileiro de Geografia e Estatística (IBGE). Ambas as bases estão disponíveis no sítio eletrônico do DATASUS do Ministério da Saúde.

Os dados foram extraídos, organizados e exportados para o aplicativo *Microsoft Excel* ^®^, no qual foram realizadas as análises descritivas. Para a análise de tendência temporal, utilizou-se o *software* Stata^®^, versão 14.0.

Foram consideradas as seguintes variáveis: sexo feminino; faixa etária igual ou superior a 60 anos; região geográfica; unidade da federação; município; ano da notificação; vínculo do agressor; local de ocorrência da violência; e ocorrência de violência de repetição.

Para o cálculo das taxas brutas de notificação, dividiu-se o número total de casos de violência por negligência e/ou abandono contra mulheres idosas pela população residente correspondente, multiplicando-se o quociente por 100.000 habitantes.

### Análise estatística e de tendência temporal

A análise da tendência temporal das taxas brutas de violência por abandono e/ou negligência contra mulheres idosas foi realizada por meio da regressão linear generalizada de Prais-Winsten, considerando nível de significância de 5% (p<0,05)^(11)^. Esse método foi adotado por permitir a correção da autocorrelação serial dos resíduos em séries temporais.

A variável dependente (Y) foi definida como a taxa bruta de notificações de violência por abandono e/ou negligência contra mulheres idosas, enquanto a variável independente (X) correspondeu ao ano da notificação. A partir do coeficiente angular (*b1*) estimado pelo modelo, calculou-se a Variação Percentual Anual (VPA), utilizada para classificar a tendência temporal como crescente (VPA positiva), decrescente (VPA negativa) ou estacionária, quando não houve diferença estatisticamente significativa em relação ao valor zero (p>0,05). A variável dependente (Y) foi a taxa bruta de violência por abandono e/ou negligência de mulheres idosas, e a variável independente (X), o ano da notificação^(11)^.

Considerando o impacto potencial da pandemia de COVID-19 sobre os registros de violência, as análises foram realizadas em dois períodos distintos -2009-2019 e 2009-2022-, permitindo avaliar a influência da inclusão desse evento no comportamento das tendências temporais. Para minimizar o viés de agregação, também conhecido como falácia ecológica, foi adotado um plano de análise estratificada segundo as grandes regiões geográficas do país e o local de residência.

Conforme o método proposto por Bottomley *et al.* ^(11)^, inicialmente, procedeu-se à transformação logarítmica na base 10 das taxas (Y), com o objetivo de reduzir a heterogeneidade da variância dos resíduos. Em seguida, aplicou-se o modelo de Prais-Winsten para estimar o coeficiente *b1*, a partir do qual foi calculada a VPA segundo a fórmula: VPA= [-1 + 10^b^_1_] *100%.

Os Intervalos de Confiança de 95% (IC95%) da VPA foram calculados utilizando os limites do coeficiente *b1* estimados pelo modelo, conforme a expressão: IC95%= [-1+10^b^_1_^mínimo^] *100; [-1+10^b^_1_^máximo^] *100. Os valores de *b*_1_ mínimo e *b*_1_ máximo são os limites do Intervalo de Confiança (IC) gerados pelo programa de análise estatística. Foram aplicados na fórmula, sendo o valor *b*_1_ mínimo correspondente ao ponto mínimo do IC, e o valor *b*_1_ máximo, correspondente ao ponto máximo do IC. Neste estudo, o nível de significância considerado foi de 5% para todos os testes estatísticos.

## RESULTADOS

A magnitude de casos de violência por negligência/abandono registrados no período de 2009 a 2022 foi de 34.860. Salienta-se que, nos anos referentes à pandemia de COVID-19, foram registrados mais de 33% dos casos de toda a série temporal. Em termos de região, a Sudeste foi a que registrou maior percentual de casos nos períodos analisados, seguida da Nordeste e da Sul.

A Tabela 1 apresenta as características das idosas vítimas desse tipo de violência de acordo com o vínculo com o agressor, local de ocorrência e violência de repetição. Entre os casos notificados, em mais de 67%, os agressores tinham vínculo familiar com a vítima. A residência foi o local de maior ocorrência da violência (>80%), denotando o abandono/negligência no próprio local de moradia, com incremento de mais de 34% da ocorrência do evento no período da pandemia de COVID-19. A violência de repetição ocorreu com mais de 40% das idosas estudadas no período.

**Tabela 1:** Caracterização das notificações de casos de violência por negligência/abandono em mulheres idosas no Brasil (2009-2022), Teresina, Piauí, Brasil, 2025

Variáveis	2009-2019		2009-2022	
	n	(%)	n	(%)
**Ocorrência da violência por negligência/abandono**				
Brasil	22.493	100	34.860	100
Norte	534	2,37	762	2,19
Nordeste	5.227	23,24	9.527	27,33
Sudeste	8.427	37,46	12.985	37,25
Sul	5.188	23,06	7.451	21,37
Centro-Oeste	3.112	13,84	4.135	11,86
**Vínculo**				
Familiar^*^	15.128	67,26	23.512	67,45
Afetivo^**^	1.145	5,09	1.682	4,83
Conhecido^***^	2.218	9,86	3.071	8,81
Desconhecido	215	0,96	298	0,85
Pessoa com relação institucional	320	1,42	476	1,37
Policial/agente da lei	15	0,07	18	0,05
Outros vínculos	3.456	15,36	5.017	14,39
**Local de ocorrência**				
Residência	18.239	81,09	27.943	80,16
Habilitação coletiva	315	1,40	505	1,45
Escola	5	0,02	7	0,02
Local de prática esportiva	9	0,04	12	0,03
Bar ou similar	4	0,02	10	0,03
Via pública	516	2,29	827	2,37
Comércio/serviços	293	1,30	515	1,48
Indústria/construção	5	0,02	7	0,02
Outros	1.186	5,27	1.834	5,26
**Violência de repetição**				
Sim	10.160	45,17	14.546	41,73
Não	4.098	18,22	7.203	20,66

* Pai, mãe, padrasto, madrasta, filho(a), irmão(a);

** Cônjuge, ex-cônjuge, namorado(a), ex-namorado(a);

*** Amigos/conhecidos, cuidador, patrão/chefe.

*Fonte: Sistema de Informação de Agravos de Notificação/Departamento de Informática do Sistema Único de Saúde.*

A Tabela 2 apresenta a análise temporal dos casos de violência por negligência/abandono de mulheres idosas no Brasil e por região. O Brasil apresentou tendência crescente estatisticamente significante para os dois períodos analisados (VPA: 20,23; IC95% 10,71; 30,57; valor de p: <0001; VPA: 16,76; IC95%: 8,62; 25,51; valor de p: <0,001). Entre as regiões, considerando o período de 2009-2019, a VPA foi maior nas regiões Norte (VPA: 43,13; IC95%: 17,73; 74,00; valor de p: 0,002) e Nordeste (VPA: 38,50; IC95%: 20,05; 59,79; valor de p: <0001). Já no período de 2009 a 2022, a região Nordeste apresentou maior VPA (VPA: 31,48; IC95%: 17,86; 46,67; valor de p: < 0,001), seguida pela região Norte (VPA: 29,58; IC95%: 8,55; 54,68; valor de p: 0,008). A região Centro-Oeste apresentou tendência estacionário para os dois períodos analisados (VPA: 8,22; IC95%: -1,82; 19,29; valor de p: 0,100; VPA: 4,47; IC95%: -3,07; 12,59; valor de p: 0,228). Não foi observada redução das taxas de violência em nenhuma região do país.

**Tabela 2:** Variação Percentual Anual das taxas brutas (por 100.000 mil/hab.) de casos de violência por negligência/abandono em mulheres idosas no Brasil (2009-2022), Teresina, Piauí, Brasil, 2025

Região	Período	VPA	IC95%	Valor de *p*	DW	Tendência
Norte	2009-2019	43,13	17,73; 74,00	0,002	1,62	Crescente
	2009-2022	29,58	8,55; 54,68	0,008	1,83	Crescente
Nordeste	2009-2019	38,50	20,05; 59,79	0,001	1,36	Crescente
	2009-2022	31,48	17,86; 46,67	< 0,001	1,33	Crescente
Sudeste	2009-2019	15,86	9,93; 22,11	< 0,001	1,61	Crescente
	2009-2022	12,72	7,21; 18,51	< 0,001	1,64	Crescente
Sul	2009-2019	30,95	9,09; 57,18	0,009	1,13	Crescente
	2009-2022	22,48	4,76; 43,19	0,015	1,14	Crescente
Centro-Oeste	2009-2019	8,22	-1,82; 19,29	0,100	1,04	Estacionária
	2009-2022	4,47	-3,07; 12,59	0,228	1,23	Estacionária
Brasil	2009-2019	20,23	10,71; 30,57	0,001	1,09	Crescente
	2009-2022	16,76	8,62; 25,51	0,001	1,17	Crescente

*VPA - Variação Percentual Anual; IC95% - Intervalo de Confiança de 95%; DW - Durbin-Watson.*

*Fonte: Sistema de Informação de Agravos de Notificação/Departamento de Informática do Sistema Único de Saúde.*

A Figura 1 mostra a evolução da taxa bruta dos casos notificados de violência por abandono/negligência em mulheres idosas por região no período de 2009 a 2022. Observa-se que, na região Centro-Oeste, a notificação dessa violência cresceu de 2009 a 2013, com período de queda e oscilações nos demais anos. A região Nordeste apresentou crescimento em todo o período, com incremento de notificações na pandemia de COVID-19. As regiões Sul e Sudeste apresentaram comportamentos semelhantes no período. A região Norte foi a que apresentou menor taxa de crescimento, com um pico de notificações entre 2015 e 2017.

**Figura 1:** Taxa bruta de casos notificados de violência por negligência/abandono em mulheres idosas no Brasil por região (2009-2022), Teresina, Piauí, Brasil, 2025

*Fonte: Sistema de Informação de Agravos de Notificação/Departamento de Informática do Sistema Único de Saúde.*

## DISCUSSÃO

Os resultados evidenciam uma tendência temporal de aumento nos casos notificados da violência por negligência/abandono da mulher idosa no Brasil, sobretudo nos anos que compreendem o ápice da pandemia de COVID-19 tanto no conjunto dos dados quanto por região do país. Apesar da magnitude do tema e das sérias consequências à saúde física e mental, a produção acadêmica ainda é incipiente em todo o mundo^(12)^. No Brasil, o Governo Federal fez o primeiro pronunciamento sobre o aumento da violência contra a pessoa idosa apenas quatro meses após o primeiro caso confirmado da doença no país^(13)^. Esse aumento pode ser atribuído a múltiplos fatores, incluindo ao isolamento social, à sobrecarga dos serviços de assistência e a uma maior vulnerabilidade socioeconômica das pessoas idosas.

Nesse cenário, é oportuno salientar que a violência contra as mulheres tende a aumentar durante emergências de qualquer natureza, incluindo as epidemias, considerando a desintegração das redes sociais e o acesso mais restrito aos serviços de saúde e de proteção social^(14)^. Embora escassos no início da pandemia de COVID-19, relatos na França, China e Estados Unidos sugeriram um aumento nos casos de violência doméstica desde o início do surto^(15)^.

Estudo realizado na África destaca o aumento do risco de violência contra a pessoa idosa por parte de seus cuidadores durante a pandemia de COVID-19^(16)^. Esse fenômeno se revela preocupante nos dados do estudo em tela, os quais remetem à ocorrência do abandono/negligência no próprio local de moradia da mulher idosa, sendo a violência praticada principalmente por pessoa com vínculo familiar.

Configura-se, assim, um cenário de violência doméstica contra a mulher idosa já evidenciado em outros estudos nacionais, mas com a sua intensificação causada pelo isolamento social decorrente das medidas de controle da contaminação pelo SARS-CoV-2. Em 2020, o jornal *The Guardian* reportou um aumento global nos casos de violência doméstica. O Brasil experimentou um aumento de 40% a 50%, e a Espanha, de 20%, no número de chamadas de apoio pelo “Disque 100” nos primeiros dias de *lockdown* ^(17)^.

Outrossim, observou-se um padrão que se repete globalmente no qual a violência contra a mulher é prevalente e perpetrada por parceiros íntimos e, no caso da pessoa idosa, por cuidadores familiares. A situação se torna ainda mais nociva quando se considera que a violência é sofrida em silêncio, e acaba sendo encoberta pela proximidade e dependência entre o agressor e a vítima ou pelo medo de retaliações e/ou abandono^(13)^.

Constatou-se, ainda, um elevado número de casos notificados de violência por repetição para os dois períodos analisados neste estudo, revelando um grave aumento na vulnerabilidade das idosas que, por vezes, são vítimas de isolamento e abandono por parte de familiares.

Esse cuidado, por vezes associado à ideia de familismo - um construto multidimensional que enfatiza a família como principal responsável pelo suporte e cuidado de seus membros -, pode se tornar um fator de vulnerabilidade quando não há um respaldo efetivo das políticas sociais e dos serviços de assistência. Tal cenário pode favorecer a ocorrência de violência intrafamiliar^(18)^.

Com isso, a dependência emocional e financeira, medo, dificuldades na utilização dos serviços sociais, e a naturalização da violência ao longo da vida contribuem para a invisibilidade dessa problemática^(10)^.

A violência e os abusos sofridos pelas pessoas idosas também podem desencadear quadros de ansiedade e sintomas depressivos, contribuindo para o isolamento social e o sentimento de solidão^(19)^. Quando essa realidade é analisada sob a perspectiva de gênero, observa-se que as mulheres idosas apresentam maior prevalência desses agravos em comparação aos homens, o que evidencia uma vulnerabilidade ainda mais acentuada, que configura como um problema de saúde pública^(4)^.

Na análise espacial, a evolução da taxa bruta dos casos notificados de violência por abandono/negligência em idosas mostra a região Nordeste do Brasil com crescimento em todo o período analisado, como apontado em resultados de estudos de séries temporais realizados anteriormente. A região Centro-Oeste apresentou oscilações no período, e a região Norte apresentou as menores taxas de crescimento. As regiões Sul e Sudeste apresentaram comportamentos semelhantes no período de análise.

O Ministério dos Direitos Humanos e da Cidadania relata que, só de janeiro a maio do ano de 2023, a Ouvidoria Nacional de Direitos Humanos por meio do “Disque 100” recebeu 47 mil denúncias sobre violência contra a pessoa idosa de todo Brasil, com registro de 282 mil violações. Nos casos de negligência e abandono, foram mencionadas 37,4 mil violações por negligência e 19,9 mil por abandono, um aumento de 855% em relação ao ano anterior^(20)^.

Ademais, o estudo desenvolvido por Camacho *et al*.^(21)^, que analisou as denúncias de violência contra pessoas idosas entre 2020 e 2023, mostrou que o maior índice por região do Brasil foi a região Sudeste, apresentando 54,63% em 2020, 54,02% em 2021, 54,38% em 2022 e 53,54% em 2023. A região Nordeste está em segundo lugar, com 20,09% das denúncias em 2020, 21,33%, em 2021, 19,93%, em 2022, e 19,90%, em 2023. Na sequência, encontram-se as regiões Centro-Oeste, Norte e, por último, a região Sul. Em todos os anos estudados, a mulher idosa se encontra sempre com o maior percentual, representando 69,22% do total vítima de violência, com maior percentual no ano de 2022.

A violência contra pessoa idosa nas regiões brasileiras não é uniforme. Devem-se considerar atores socioeconômicos, culturais e de acesso a serviços de proteção, que variam entre as regiões, impactando a prevalência e a tipologia das agressões. Nesse contexto, Andrade *et al*.^(22)^ investigaram esse perfil na perspectiva da urgência e emergência por meio do VIVA Inquérito de 2017. O estudo identificou que o sexo masculino obteve o maior índice de agressão física por um desconhecido. Já entre as idosas, os índices destacaram a violência praticada pelo companheiro, sendo a ameaça uma das formas mais recorrentes de agressão.

Dessa forma, é necessário que as políticas públicas sejam ajustadas às especificidades regionais, considerando fatores como infraestrutura, acesso a serviços de saúde e vulnerabilidades socioeconômicas, para combater e prevenir a violência contra a pessoa idosa. Além disso, é necessário combater o idadismo e o machismo, quando equiparados diretamente às mulheres idosas, tendo em vista que o gênero é um fator de risco considerável^(23,24)^.

Diante desse cenário, recomenda-se a ampliação de estratégias de enfrentamento da violência contra a mulher idosa, incluindo campanhas de conscientização, aprimoramento da formação de profissionais da saúde e assistência social, e fortalecimento dos canais de denúncia e suporte. Estudos futuros podem aprofundar a análise de determinantes sociais e fatores psicossociais que contribuem para a perpetuação desse tipo de violência, além de explorar intervenções eficazes para sua prevenção e mitigação.

### Limitações do estudo

A limitação se refere à possibilidade de subnotificação dos casos de violência por negligência/abandono, especialmente nas regiões Norte e Nordeste do Brasil. Esse fator pode comprometer a acurácia dos dados e limitar a generalização dos resultados. Por se tratar de dados secundários, há dependência da completude e consistência do preenchimento no sistema de notificação. Além disso, a categorização da violência por negligência e abandono pode não abranger toda a complexidade das interações sociais e culturais que permeiam essa forma de violência.

### Contribuições para a área da enfermagem, saúde ou políticas públicas

Este estudo amplia a compreensão sobre a distribuição regional e os padrões de negligência contra mulheres idosas, oferecendo suporte técnico-científico para que profissionais de enfermagem planejem estratégias de cuidado mais sensíveis às situações de vulnerabilidade. Em termos de saúde coletiva, destaca a necessidade de reforçar a formação permanente voltada à detecção precoce e ao enfrentamento da violência.

No campo das políticas públicas, os achados reforçam a urgência de desenvolver ações intersetoriais que priorizem a segurança, o cuidado e os direitos da população idosa no contexto domiciliar, tais como a capacitação contínua de cuidadores e de familiares para o cuidado domiciliar, o fortalecimento dos centros de referência, e a ampliação de redes comunitárias e intergeracionais de apoio voltadas à criação de vínculos protetores e à redução do isolamento das pessoas idosas. Tais estratégias favorecem a implementação de políticas de envelhecimento ativo, participativo e seguro.

Com isso, a pesquisa contribui para o entendimento da violência contra mulheres idosas no Brasil. A crescente notificação desses casos ressalta a urgência de ações concretas que garantam o direito à dignidade, segurança e qualidade de vida para essa população.

## CONCLUSÕES

O presente estudo alcançou seu objetivo ao demonstrar tendência temporal crescente das notificações de violência por negligência e abandono contra mulheres idosas no Brasil entre 2009 e 2022, com destaque para o período da pandemia de COVID-19. Constatou-se um número absoluto maior de registros na região Sudeste e um VPA maior no Nordeste, o que evidencia desigualdades regionais marcantes.

A predominância dos casos no ambiente domiciliar e o envolvimento de familiares como principais agressores revelam o caráter silencioso e persistente dessa forma de violência, frequentemente invisibilizada pelos serviços de saúde. Tais achados reforçam a necessidade de fortalecimento das ações de vigilância, detecção precoce e acompanhamento contínuo no âmbito da Atenção Primária à Saúde, com protagonismo da enfermagem no cuidado e na escuta qualificada das famílias.

Recomendam-se a ampliação das estratégias de capacitação de cuidadores e profissionais de saúde, o aprimoramento dos fluxos de notificação e o fortalecimento da articulação entre os serviços de saúde, assistência social e justiça. Essas ações são fundamentais para o enfrentamento efetivo da negligência e do abandono, bem como para a consolidação de políticas públicas voltadas à promoção de um envelhecimento ativo, digno e protegido, em consonância com as diretrizes da Década do Envelhecimento Saudável nas Américas (2021-2030).

## Data Availability

Os dados de pesquisa só estão disponíveis mediante solicitação.
